# Evaluation of GoGirlGo!; A practitioner based program to improve physical activity

**DOI:** 10.1186/1471-2458-14-118

**Published:** 2014-02-05

**Authors:** Jennifer L Huberty, Danae M Dinkel, Michael W Beets

**Affiliations:** 1 Exercise and Wellness, School of Nutrition and Health Promotion, Arizona State University, 500 N 3rd, St. Phoenix, AZ 85004, USA; 2 School of Health, Physical Education, and Recreation, University of Nebraska Omaha, 6001 Dodge Street, Omaha, NE 68182, USA; 3 Division of Health Aspects of Physical Activity, Department of Exercise Science, Arnold School of Public Health, University of South Carolina, 921 Assembly St., 1st Fl. Suite, RM 131, Columbia, SC 29208, USA

## Abstract

**Background:**

GoGirlGo! (GGG) is designed to increase girls’ physical activity (PA) using a health behavior and PA-based curriculum and is widely available for free to afterschool programs across the nation. However, GGG has not been formally evaluated. The purpose of this pilot study was to evaluate the effectiveness of the GGG curricula to improve PA, and self-efficacy for and enjoyment of PA in elementary aged girls (i.e., 5-13 years).

**Methods:**

Nine afterschool programs were recruited to participate in the pilot (within subjects repeated measures design). GGG is a 12-week program, with a once a week, one-hour lesson with 30 minutes of education and 30 minutes of PA). Data collection occurred at baseline, mid (twice), post, and at follow-up (3-months after the intervention ended). PA was assessed via accelerometry at each time point. Self-efficacy for and enjoyment of PA was measured using the Self-Efficacy Scale and the Short-PA enjoyment scale and was assessed at baseline, post, and follow-up. Fidelity was assessed at midpoint.

**Results:**

Across all age groups there was a statistically significant increase in PA. Overall, on days GGG was offered girls accumulated an average of 11 minutes of moderate-to-vigorous PA compared to 8 minutes during non-GGG days. There was a statistically significant difference in girls’ self-efficacy for PA reported between baseline and post, which was maintained at follow-up. An improvement in enjoyment of PA for girls was found between baseline and follow-up. According to fidelity assessment, 89% of the activities within the curriculum were completed each lesson. Girls appeared to respond well to the curriculum but girls 5-7 years had difficulties paying attention and understanding discussion questions.

**Conclusions:**

Even though there were statistically significant differences in self-efficacy for PA and enjoyment of PA, minimal increases in girls’ PA were observed. GGG curricula improvements are warranted. Future GGG programming should explore offering GGG every day, modifying activities so that they are moderate-to-vigorous in intensity, and providing additional trainings that allow staff to better implement PA and improve behavior management techniques. With modifications, GGG could provide a promising no-cost curriculum that afterschool programs may implement to help girls achieve recommendations for PA.

## Background

Girls are consistently less physically active than boys regardless of race, income level, weight status, age, and/or setting (i.e., before, during or after school) [1,2]. Only 35% of girls (6–11 years) achieve the recommended 60 minutes of daily moderate-to-vigorous physical activity (MVPA) compared to 48% of boys [3,4]. Girls repeatedly report higher levels of interest in sedentary leisure pursuits such as board games and talking with friends as compared to boys [5-7]. Girls also do not participate in as much physical activity (PA) as boys due to concerns about their appearance, being self-conscious in front of peers, and lack of interest [5,6,8,9]. Further, girl’s PA levels decline more rapidly and at a younger age than boys [1,10]. This lack of PA is related to increased risk for type 2 diabetes, elevated cholesterol and high blood pressure in youth [2]. Because low levels of PA persist into adolescence and adulthood [7], there is also an increased risk for chronic disease (i.e., heart disease, diabetes, obesity, cancer) later in life [11,12]. Childhood, therefore, represents a critical time to promote girls’ PA.

Few interventions have been successful at substantially increasing MVPA in girls. In interventions targeting boys and girls, boys often show greater improvements in PA as compared to girls [13-16]. For example, in a study to improve MVPA during recess, boys’ MVPA increased by 19% compared to controls while girls’ MVPA increased by only 6% compared to controls [3]. Regrettably, interventions that specifically target girls are limited and have not been any more successful at improving girls’ MVPA than studies targeting both boys and girls. This includes large-scale, randomized controlled trials such as the Girls Health Enrichment Multi-site Studies (GEMS) [17,18] and the Trial of Activity for Adolescent Girls (TAAG) [19]. The lack of success in improving girls PA represents a considerable challenge that needs to be addressed.

One way in which to improve PA in girls may be to assure that programs/curricula that are designed and used by practitioners within real-world settings (i.e., not being implemented for research purposes) are effective. Interventions targeting girls that are designed for research purposes often lack components to support real-world sustainability. For example, GEMS, a multi-site trial to prevent weight gain in 8–10 year-old African American girls, was developed for research purposes and then applied to afterschool programs (ASPs) in a variety of formats. After the intervention, investigators reported that continued enthusiasm and participation from girls and their parents would be a significant challenge due to the unrealistic time commitment required outside of the intervention setting [17]. Additionally, researchers recommended that future research should consider enhancing existing programs or environments to improve health behaviors in girls, as opposed to creating new programs [18]. The TAAG intervention provided further evidence for the potential positive impact afterschool time (2-5 pm) may have on children’s PA but more research in this area is warranted.

While there are numerous programs available for practitioners, one promising and widely disseminated program is GoGirlGo! (GGG). Developed by the Women’s Sports Foundation, GGG has been offered in a number of urban afterschool settings (e.g., Boys and Girls Club afterschool youth service agency) and is designed to improve the health of sedentary girls ages 5–13 years and keep girls involved in PA. GGG provides separate developmentally appropriate curriculum for 5–7, 8–10, and 11–13 year old girls which includes PA opportunities and weekly discussions about various health-related behaviors (e.g., building confidence, bullying, diversity, and PA). Despite its reach (1,000,000 girls across the U.S.) and national presence (over 15,000 organizations receiving the free curriculum), GGG has not been evaluated for its effectiveness in increasing PA. Therefore, the purpose of this pilot study was to evaluate the effectiveness of the GGG curricula to improve PA, self-efficacy for PA, and enjoyment of PA in elementary aged girls.

## Methods

### Study design and participants

This pilot study was approved by the University of Nebraska Medical Center Institutional Review Board in the Midwestern United States. Using a within subjects repeated measures design, nine ASPs from the Midwest region were recruited to pilot the GGG curriculum in girls ages 5–7, 8–10, and 11–13 years. ASP organizations who primarily worked with underserved populations (e.g., racial/ethnic minorities) were identified through the PIs existing relationships and an online search for ASPs in the community. Research personnel then contacted the organizational directors to determine their interest in the study. Sites were recruited until the potential to achieve the total target sample size within sites was reached (n = 50 participants in each age group). Once a site director agreed to participate, research personnel attended the site to explain the study to the girls at the ASP. All girls at the ASPs (n = 305) were invited to participate in the study and were asked to return a signed parental consent form. Additionally, a brief demographic survey (birth date and race/ethnicity) was attached to each consent form and was completed by girls’ parents/guardians. The study was conducted between August 2012 and December 2012 with follow-up in March of 2013. Baseline evaluation occurred prior to implementation of GGG (August-September), mid-evaluation was conducted twice during implementation of GGG (September-November) and post evaluation was conducted one week after GGG ended (December). Follow-up was conducted three months after the intervention ended (March).

### GGG! Curricula

All of the participating sites offered GGG one day a week for one hour. Each class focused on a specific developmentally appropriate life skills topic (e.g., bullying, body image). Thirty minutes of class consisted of reading stories about a champion female athlete or peer role model who had personally experienced the life skills topic (i.e., being bullied) and group discussion related to that topic. The remaining 30 minutes was spent participating in PA intended to reinforce the topic covered that day. For example, the PA for the lesson on bullying for 8–10 year old girls consisted of splitting the girls into two teams with one group being much larger than the other group. The girls then played tug-of-war with the larger side representing bullies. The teams played for a few minutes and then the instructor had girls from the bullying side move over to the other side to symbolize how girls can help each other out in these situations. Each lesson is intended to introduce girls to fun PA and at the same time, engage girls in honest conversations with a trusted adult leader about social and health risks. See Table 1 for examples of lesson topics and corresponding activities for the 8–10 year olds. Prior to implementing GGG, existing staff who were identified by site directors at the ASP attended a one-hour training. The training consisted of a brief explanation of the purpose and history of GGG, discussion on facilitation tips (e.g., making it a safe environment for girls to share their feelings), an overview of the curriculum and materials (e.g., leader guide for staff, journals for girls), and practicing one of the lessons. After the training, ASP staff were provided with a leader guide that included the lesson topics and physical activities for each of the 12 weeks for their respective age group. Those staff teaching the 8-10 and 11-13 year old curriculum were also provided with scrapbooks/journals for the girls that included the stories of athletes and pages to write about their thoughts and feelings. ASP staff and their site directors were expected to follow the leader guide and encouraged to contact research personnel if needed. This specific training is similar to what GGG provides to organizations when there is funding available. Typically, when organizations request the GGG curriculum from the Women’s Sports Foundation, no training is provided.

**Table 1:** Lesson topics and corresponding PA for 8–10 year old curriculum

**Week**	Topic*	**PA**
1	Building confidence	PA assessment – Girls perform a warm-up followed by a variety of activities (e.g., tuck jumps, push-ups, mountain climbers). Girls measure their heart rate before and after the activities.
2	Dealing with difficult feelings	Green light/Red light – Girls play the traditional game and if they are caught moving, they must give an example of how someone could deal with being angry or upset.
3	Nutrition	Healthy meal roundup – Girls are given a name tag with the name of a food on it. Girls run around the room until leader says stop and tells them to pair up with another type of food
4	Smoking/Substance abuse	Freeze skate – Girls practice speed skating with their socks on
5	Body image	Track and field activities – Girls practice long jump, triple jump, and high jump.
6	Self-care	Charades – Girls run to different signs with types of self-care actions and act them out.
7	Teamwork/Cooperation	Partner “races” – Girls participate in various forms of races with their partner (e.g., 3-legged race).
8	Playing fair	Team race – Girls work together to move a ball from one end of the room to the other using various methods (e.g., carrying, throwing).
9	Diversity	Huddle up – Girls jog around the room until the leader yells out an instruction to get into a group (e.g., groups of four) and a type of activity to perform. Girls then get in a group and perform that activity while answering a question within the group.
10	Bullying	Tug-of-War – Girls play tug-of-war with different sides representing bullies and defenders.
11	Community service	Graffiti Laps – Girls run around the room until the leader says to stop, then they write answers to questions about their community.
12	Strong body/Strong mind	PA assessment – Girls perform a warm-up followed by a variety of activities (e.g., tuck jumps, push-ups, mountain climbers). Girls measure their heart rate before and after the activities.

*Note each age group curricula is different and this represents the 8–10 year old curriculum.

### Outcome measures

Girls that returned a signed parental consent were assessed for Body Mass Index at baseline. Trained graduate assistants took anthropometry measurements from every participating girl. Girls wore one layer of clothing and removed their shoes for both measurements. Height was measured to the nearest 1/8 of an inch using a SECA stadiometer and weight was measured to the nearest tenth of a pound using an electronically calibrated digital scale. Outcome measures included PA, self-efficacy for PA, and enjoyment for PA. PA was measured objectively using Actigraph uniaxial accelerometers (GT1M, Actigraph LLC, Pensacola, FL). The Actigraph is a small monitor designed to detect vertical accelerations and is a valid and reliable instrument to measure PA in elementary aged children [20]. Five second epochs were used to capture girls’ PA and distilled into time spent sedentary, light, and MVPA according to established cutpoints [20]. The primary outcome was time spent in MVPA. Girls wore the accelerometers during the entire time in attendance at the ASPs. Research personnel placed the accelerometers on the right hip of girls as they entered the ASP and retrieved them just before the girls left for the evening. This was repeated during each of the four consecutive data collection days at baseline, post, and follow-up. Research personnel documented the time each accelerometer was placed on and taken off each child. Total time in attendance was computed as time off minus time on. At mid-evaluation devices were worn during GGG days and non-GGG days. This was designed to compare PA levels when GGG was delivered vs. days during the same week when GGG was not implemented. On non-GGG days girls participated in their normal ASP activities which may have included the following: educational games, tutoring, reading activities, PA, and/or arts and crafts. Only girls that attended an ASP for at least 60 minutes each day were included in the final analyses [4,21,22].

Self-efficacy for PA and enjoyment of PA was measured using the Self-Efficacy Scale [23] and the Short-PA Enjoyment Scale, respectively [24,25] at baseline, post, and follow-up. The self-report instruments were given to girls at each site in a classroom setting. Research personnel explained how to complete each instrument and answered questions as necessary. Both of these self-report instruments have been widely used in school-aged children and are reliable and valid tools in this population [26,27].

The research team conducted one unannounced fidelity check per site with each age group at midpoint at each site. Research personnel attended each site to observe staff while they taught a lesson and documented items related to program implementation. The checklist for each lesson was created and included the following: 1) Staff implementation of the lesson (i.e., followed content according to the leader guide), 2) Time spent in education (i.e., stories and group discussion) and PA, 3) Time spent in lecture (i.e., staff reading or explaining the topic), discussion, demonstration (i.e., showing girls an activity), and practice (i.e., actually participating in PA), 4) Children’s response to the curriculum (i.e., rated on scale of 1 (low) to 5 (high)), and 5) Ability of instructor to teach curriculum (i.e., rated on scale of 1 (low) to 5 (high)). Also, research personnel documented aspects of the curriculum that appeared to work well or that did not work well.

### Statistical analysis

Descriptive statistics were used to describe the main characteristics of the group. Initially, changes in total PA (light to vigorous) and MVPA were evaluated from baseline to follow-up assessments on non-GGG days. This analysis was performed to determine whether changes in routine practice, which may lead to changes in girls’ activity levels, occurred outside the days when GGG was delivered. Secondly, a comparison of activity levels between GGG and non-GGG days was performed to evaluate the impact of GGG on girls’ activity. Finally, comparison of activity levels between GGG and non-GGG days was performed for each age group (i.e., 5-7 years, 8-10 years, 11-13 years). All models controlled for the time varying total time in attendance at the afterschool program for each girl on each day of measurement. Mixed effects repeated measure ANOVA models accounting for multiple days of accelerometer measure nested within girls nested within ASPs were used for these analyses. Mean and standard deviation scores were calculated for self-efficacy for PA and enjoyment of PA. Differences over time were evaluated using a related samples Friedman test. Mean scores for each aspect of the fidelity checklist were also calculated. All analyses were conducted using Stata (v.12.0, College Station, TX).

## Results

### Study participants

**Figure 1:**
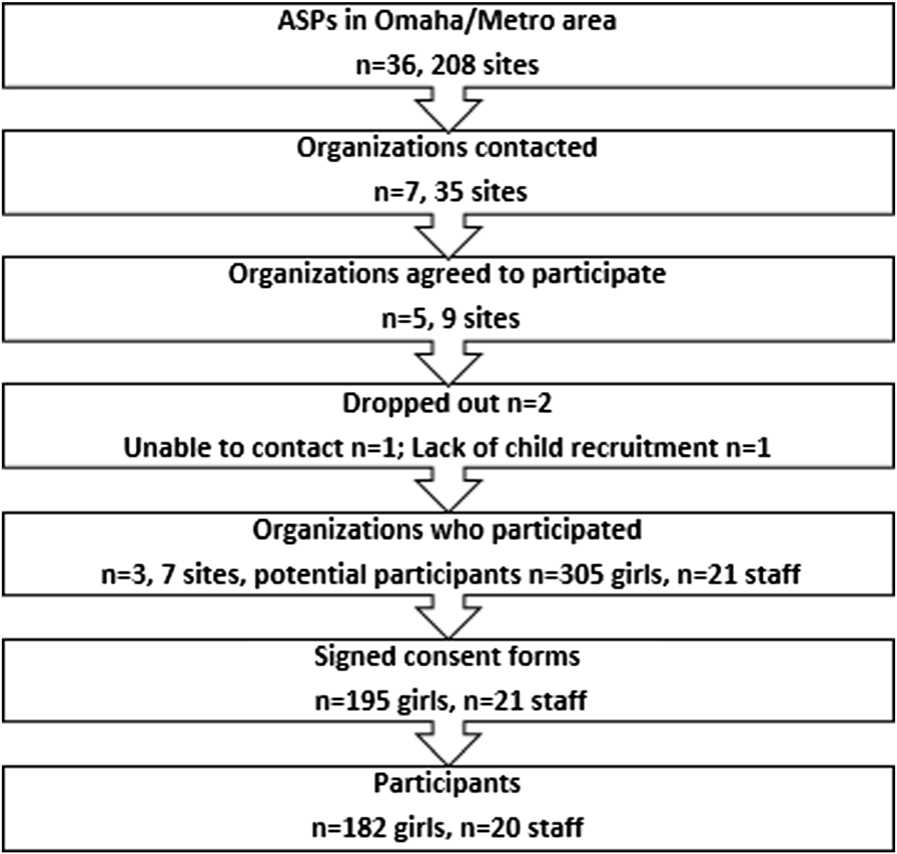
GGG Recruitment.

Recruitment of the nine ASPs and participants are illustrated in Figure 1. Two of the nine ASPs had implemented an older version (2009) of GGG previously. The number of sessions offered at each site, for each age group, is provided in Table 2. Of the 305 girls invited to participate, 182 returned signed informed consents. The 182 girls represented a total of 1,533 measurement days with a minimum of 60 minutes of wear time. Each girl was measured an average of eight measurement days across the entire study.

**Table 2:** Number of GGG chapters completed by site and age group

**Site**	**Age group**	**GGG chapters completed (of 12)**
1	8-10	12
	11-13	12
2	8-10	9
3	5-7	12
	8-10	12
	11-13	11
4	5-7	12
	8-10	12
5	5-7	12
	8-10	8
	11-13	12
6	5-7	12
	8-10	12
7	8-10	6

Approximately half of the girls were overweight (25.3%) or obese (24.7%) and a majority of the girls were either Black (43.9%) or Hispanic (34.6%). See child demographics in Table 3.

**Table 3:** Child demographics

	**5-7 yrs**			**8-10 yrs**			**11-13 yrs**		
**Variable**	***n***	**M**	**SD**	***n***	**M**	**SD**	***n***	**M**	**SD**
Age (years)	33	6.4	0.7	90	9.2	0.8	59	11.3	0.7
Body mass index	29			90			55		
Normal weight		58.6%			32.4%			26.8%	
Overweight		17.2%			44.1%			46.7%	
Obese		24.1%			23.5%			26.5%	
Race/ethnicity	32			87			58		
Black		53.0%			48.9%			47.3%	
Hispanic		36.0%			27.8%			25.5%	
Other		11.0%			23.3%			27.3%	

### PA

**Figure 2:**
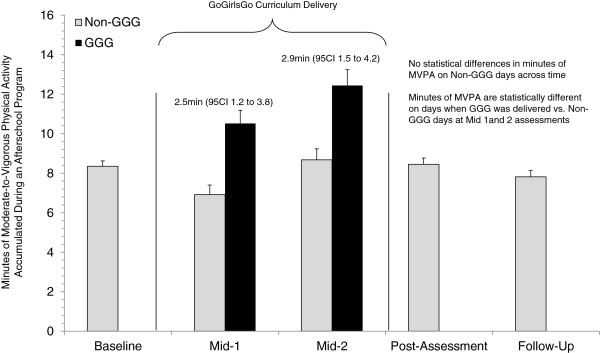
Estimates of MVPA during GGG and non-GGG days from baseline to follow-up.

**Figure 3:**
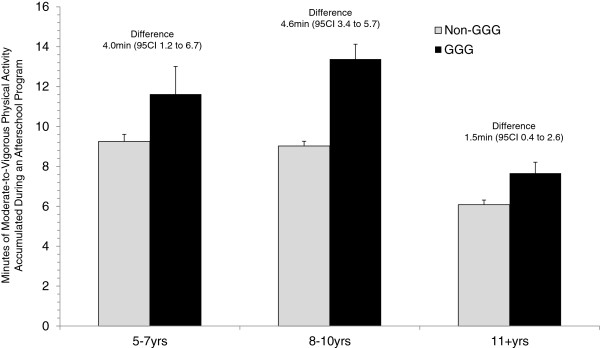
Comparison of moderate-to-vigorous physical activity during GGG and non-GGG days among three age groups.

**Figure 4:**
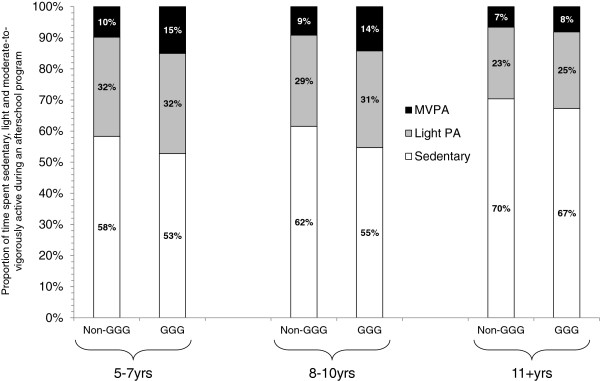
Time spent sedentary, light PA and MVPA during GGG and non-GGG days by age groups.

The time spent sedentary and physically active across each time point is presented in Table 4. There were no differences in the amount of MVPA girls accumulated on non-GGG days from baseline to follow-up, indicating girls activity levels were stable from baseline to follow-up (see Figure 2). Comparisons between GGG and non-GGG days at midpoint 1 and 2 indicated that, for the overall sample of girls, on days when GGG was delivered an increase in 2.5 and 2.9 minutes of MVPA occurred. Differences in accumulated MVPA on GGG and non-GGG days for each age group are presented in Figure 3. Across each of the three age groups, GGG was associated with a statistically significant increase in MVPA, with the largest difference for the 8-10 year olds (4.6 min, 95CI 3.4 to 5.7), followed by the 5-7 year olds (4.6 min, 95CI 3.4 to 5.7), and finally the 11-13 year olds (1.5 min, 95CI 0.4 to 2.6). Differences in the percentage of time spent at each intensity (i.e., sedentary, light, and moderate-to-vigorous) between GGG and non-GGG days for each age group are presented in Figure 4. For both the 5-7 year-olds and 8-10 year-olds, the increase in MVPA (+5%) occurred predominately from replacing time spent sedentary (58% vs. 53% and 62% vs. 55%, respectively).

**Table 4:** Time spent in physical activity and sedentary during GGG and non- GGG days

			**Mid 1 (avg 5.7wks, SD ± 1.1)**^**a**^				**Mid 2 (avg 2.7wks, SD ± 1.8)**^**a**^							
	**Baseline**		**Non-GGG**		**GGG**		**Non-GGG**		**GGG**		**Post-Assessment (avg. 3.0wks, SD ± 1.3)**^**a**^		**Follow-up (avg. 14.0wks, SD ± 1.8)**^**a**^	
**Activity variable**	**M**	**SD**	**M**	**SD**	**M**	**SD**	**M**	**SD**	**M**	**SD**	**M**	**SD**	**M**	**SD**
Sedentary	68.3	±29.6	50.7	±26.3	50.5	±16.4	54.4	±24.7	57.4	±20.1	62.8	±29.7	53.5	±22.3
Physical activity														
Light	26.4	±15.5	24.1	±15.7	25.8	±10.1	25.7	±13.7	28.1	±10.6	28.5	±15.0	26.4	±13.7
Moderate	4.7	±3.4	4.2	±3.4	5.6	±3.5	4.8	±3.6	6.8	±3.9	5.2	±3.5	4.6	±3.3
Vigorous	3.6	±3.4	2.8	±2.8	4.9	±3.0	3.9	±3.5	5.7	±3.7	3.3	±3.0	3.2	±3.0
Moderate-to-vigorous	8.4	±6.5	6.9	±6.0	10.5	±6.1	8.7	±6.9	12.4	±7.2	8.5	±6.1	7.8	±6.1
Total time in attendance^b^	103.0	±39.5	81.8	±40.1	86.8	±21.8	88.8	±36.6	98.0	±25.8	99.8	±42.8	87.7	±31.8

Abbreviations: GGG = GoGirlGo!

^a^Average number weeks between assessments. Example, an average of 5.7wks elapsed from baseline to the first Mid point1 assessment; an average of 14wks elapsed from post-assessment to follow-up.

^b^Based on total accelerometer wear time from the start of the program to the time a child leaves the program.

### Self-Efficacy and Enjoyment for PA

There was a statistically significant increase in self-efficacy for PA from baseline to post (p < .001), and baseline to follow-up (p < .001). These findings indicate that when asking questions such as “Can you do PA most days of the week?” girls were more likely to mark “yes” compared to “no”. There were no differences in enjoyment for PA between baseline and post-intervention but there was a statistically significant difference between baseline and follow-up (p = .016). This indicates that girls became more likely to report they “disagree a little” compared to “neither disagree or agree” to statements such as “When I am active it’s no fun at all.” See Table 5.

**Table 5:** Self-efficacy for PA and enjoyment of PA assessment

	**Baseline**			**Post**			**Follow-up**		
	**n**	**M**	**SD**	**n**	**M**	**SD**	**n**	**M**	**SD**
Self-efficacy for PA	139	.41	.23	99	.63**	.33	86	.64**	.38
Enjoyment for PA	139	3.8	1.03	100	4.0	.93	88	4.2*	.77

Note: Questionnaires were completed by girls eight years and older.

*Statistically significant p < .05 compared to baseline measure.

**Statistically significant p < .001 compared to baseline measure.

### Fidelity

Seventeen of the 20 staff members who taught at least one lesson at participating sites were observed. In one instance, it was discovered that a staff member who was not trained taught a GGG lesson and did not follow the curriculum. The research team spoke with the directors of the site and they agreed to not have her teach a lesson on her own moving forward. Approximately 89% of the activities within the curriculum were completed during each lesson. Most of the education curriculum was spent in discussion (46%) and lecture (32%). The PA curriculum was most often spent in practice (52%) and discussion (21%). Approximately 28 minutes were spent conducting the education curriculum and 23 minutes were spent conducting the PA curriculum. Girls appeared to respond well (e.g., engaged in discussion and PA) to the curriculum (score of 4 on scale of 1 to 5) with lowest scores coming from 5-7 year-old responses to the education (3.3) and 8-10 year-olds response to the PA (3.2). See Table 6.

**Table 6:** Implementation fidelity

**Variable**	**All Ages**	**5-7**	**8-10**	**11-13**
% Curriculum implemented*	89.8%	93.8%	84.2%	91.47%
During education curriculum the% time instructor spent in:				
Lecture	32.0%	44.0%	27.0%	23.0%
Discussion	46.0%	43.0%	47.0%	49.0%
Demonstration	8.3%	4.4%	11.0%	9.7%
Practice	11.0%	4.4%	12.0%	17.0%
During PA curriculum the% time instructor spent in:				
Lecture	15.9%	16.9%	13.5%	17.3%
Discussion	21.0%	13.0%	16.0%	34.0%
Demonstration	9.3%	3.8%	11.0%	13.0%
Practice	52.0%	66.0%	57.0%	33.0%
Time spent in education (minutes)	28.1	23.9	32.8	27.8
Time spent in PA (minutes)	23.7	20.2	29.5	21.5
Girls response to education (1(low) to 5(high))	3.9	3.3	3.9	4.4
Girls response to PA	3.8	4	3.2	4.2
How well instructor taught education (1(low) to 5(high))	4.1	4	4.1	4.1
How well instructor taught PA	3.9	3.9	3.9	3.9

*This is the percentage of curriculum implemented only during fidelity checks.

The research team documented problems staff had delivering the curriculum or changes staff made to the curriculum. Girls between 5-7 years of age were not able to sit still very long, had difficulty listening to the stories, were easily distracted, and did not always seem to understand the discussion questions. Several staff also were observed having difficulty adjusting activities for the girls ages 5-7 years. Classroom management was a challenge for staff in all age groups with staff having to stop lessons to manage behaviors or discipline. At three of the sites, girls (8 years old and above) and/or staff were so interested in the discussion that minimal time was spent participating in PA. Sites were also not fully utilizing the scrapbook and journals; four of the seven sites did not consistently hand out the scrapbooks/journals to the girls.

## Discussion

The purpose of this pilot study was to evaluate the effectiveness of GGG, a nationally recognized curriculum, implemented in a real-world setting (ASPs) to improve PA, self-efficacy for PA, and enjoyment of PA in elementary aged girls. The findings from this pilot study represent the first scientific evaluation of GGG. There was an increase in PA afterschool during days that the GGG curriculum was implemented as well as increases in self-efficacy for and enjoyment of PA. Despite these increases, the overall impact on MVPA was minimal, yielding 11 minutes of MVPA on GGG days as compared to 8 minutes of MVPA on non-GGG days. Notably, increases in MVPA were not sustained when GGG was not implemented.

Because one of the strengths of GGG lies in its ability to reach a large, diverse population of girls, it is important that the curriculum contributes to girls’ achievement of 60 minutes of daily MVPA. Modifications of the GGG curriculum are therefore warranted. GGG was originally designed to offer PA one day a week. Research suggests that PA should be offered daily in the afterschool setting because afterschool may be the only opportunity during the day that girls’ are provided activity [4], and girls may need more time to accumulate the same levels of MVPA as boys [16]. Furthermore, the activities offered in GGG, may not support MVPA. Tug-of-war and team relay activities, while helping to reinforce educational topics, may not allow a majority of girls to accumulate higher intensity PA. Additionally, the GGG curriculum has discussion woven into PA, further reducing the amount of time available for MVPA. Our fidelity assessment suggested that 21% of the time allotted for PA on the one day per week that GGG was offered was spent in discussion. This means that in addition to GGG only being implemented once a week, over six of 30 allotted minutes of PA available are being lost to inactive time.

Modifications to enhance GGG to assure girls achieve recommended daily MVPA, may include: (1) use moderate-to-vigorous type activity to correspond with lesson topics (as compared to the activities currently within the curriculum), (2) offer GGG every day, and (3) limit discussion to educational time only as compared to discussion being conducted in both educational and PA time. For example, instead of tug-of-war (lower intensity activity) as a means to illustrate bullying, staff may implement a game of tag in which several girls are the taggers (bullies). Once a girl is tagged she must do jumping jacks (or similar activity) until tagged by the defender (defender allows girls back into the game). Group-based tag allows girls to accumulate MVPA, as compared to less intensity activity (e.g. tug-of-war) or standing still, to reinforce the topic of bullying. Furthermore, these activities can be implemented throughout the week to provide daily opportunities to achieve MVPA (as compared to one day per week). Finally, discussion about bullying would only take place during the time allotted for education, not the time allotted for PA.

The staff-training component of GGG may also need to be modified to assure that staff can maximize time spent in activity during GGG. Weaver and colleagues [28] have suggested competency-based staff training (i.e., 5 M’s; Mission, Motivate, Monitor, Manage, Maximize), in which staff are trained on the necessary skills (e.g., facilitating small sided games, eliminating lines while children are waiting for an activity) to maximize PA time in any environment, using any curriculum. Additionally, McKenzie and colleagues [29] have long suggested that staff professional development training is needed to ensure quality and quantity of instruction that maximizes activity time. In our study, younger girls (5-7 years) were bored easily with the stories and had difficulty answering the discussion questions. Further, staff may have been challenged with adapting PA for the younger girls. Several staff had difficulties with classroom management and often had to stop lessons for behavior issues. Research personnel noted that more girls were engaged in PA when staff supplemented GGG PA with an activity they knew was popular with the younger girls (e.g., freeze tag) or made adaptations to GGG curriculum activities. These staff may have had more training or experience prior to GGG related to age-appropriate encouragement of PA. Training staff on age-appropriate behavior management techniques and supplemental PA could positively increase girls MVPA during GGG. This provides further justification for an enhanced version of GGG.

According to our fidelity assessments, girls 8 years and older responded well to the educational curriculum (i.e., discussions on bullying, body image). The positive response to the curriculum and the minimal yet significant improvements in self-efficacy are important. The educational portion of GGG in which principles of self-efficacy of PA are taught, is a unique aspect of this practitioner-based curriculum. GGG uses education and discussion to help girls develop positive relationships with PA, themselves, and other girls. This could have a positive impact on girls’ emotional/psychological health in the future regardless of their PA participation,

### Limitations

Despite the strengths of this pilot study there were a few limitations. First, while we did obtain consent for 182 girls, it was difficult to get children to return signed consent forms (305 available girls attending the ASPs), limiting our total sample size and thus representativeness of the sample. Second, this study was not an RCT and did not include a control group, limiting the conclusions regarding effectiveness. However, we utilized a case-crossover design in which within-subjects effects were tested across repeated measures. This design allowed us to evaluate changes in MVPA in the same girls under two different conditions – GGG and non-GGG days. Third, although staff were asked to complete attendance each time GGG was offered, staff did not complete attendance logs. This is a limitation, as we don’t know how many girls attended each session. Even though fidelity checks were unannounced, the presence of research personnel may have influenced the quality of the program on that specific day. Additionally, fidelity checks were only conducted once per session and may not be a true representation of the fidelity of the program.

## Conclusion

This study was the first scientific evaluation of GGG, a free, nationally recognized curriculum for girls. Although there are many programs available for use by practitioners in real-world settings, few, if any, have been evaluated for their effectiveness to improve PA, especially in girls. Although there were increases in MVPA, self-efficacy for and enjoyment of PA as a result of GGG, these changes were minimal. Our findings provide data that will guide enhancements to GGG. This is important as GGG has impacted over 1,000,000 girls to date and its reach continues to grow. Future modification to GGG related to PA may include offering GGG on all days of the week and specific staff training/materials about how to manage PA time and behavioral issues, and how to better engage young girls in the education curriculum. With modifications, GGG may provide a promising curriculum that ASPs can implement to help assure that girls are achieving recommendations for PA.

## Abbreviations

PA: Physical activity; ASP: Afterschool program.

## Competing interests

The authors declare they have no competing interests.

## Authors’ contributions

JH contributed to the design of the study, managed the study, participated in data collection, and drafted the manuscript. DD contributed to the design of the study, participated in data collection, analyzed data, and reviewed and commented on the manuscript. MB contributed to the design of the study, analyzed data, and reviewed and commented on the manuscript. All authors read and approved the final manuscript.

## Pre-publication history

The pre-publication history for this paper can be accessed here:

http://www.biomedcentral.com/1471-2458/14/118/prepub
